# Targeting emerging amino acid dependencies and transporters in cancer therapy

**DOI:** 10.3389/fphar.2025.1717414

**Published:** 2025-11-19

**Authors:** Alfred Akinlalu, Emmanuel Ogberefor, Tommy Gao, Dali Sun

**Affiliations:** 1 Biomedical focus, Department of Electrical and Computer Engineering, University of Denver, Denver, CO, United States; 2 Knoebel Institute of Healthy Aging, University of Denver, Denver, CO, United States; 3 Center for Advanced Biosensing Engineering (CABE), Deparment of Electrical and Computer Engineering, University of Denver, Denver, CO, United States

**Keywords:** amino acid metabolism, cancer therapy, nutritional intervention, dietary modulation, metabolic targeting, amino acid transporters, glutaminase inhibition, SLC transporters

## Abstract

Amino acid metabolism is an important vulnerability in cancer. Established strategies such as arginine depletion, glutaminase inhibition, tryptophan-kynurenine modulation, and methionine restriction have shown that these pathways can be targeted in patients. At the same time, clinical trials reveal two consistent challenges: tumors can adapt by redirecting their metabolism, and reliable biomarkers are needed to identify patients who are most likely to benefit. Recent studies point to additional amino acids with translational potential. In pancreatic cancer, histidine and isoleucine supplementation has been shown in preclinical models to be selectively cytotoxic to tumor cells while sparing normal counterparts. In glioblastoma, threonine codon-biased protein synthesis programs that support growth; in other contexts, lysine breakdown suppresses interferon signaling through changes in chromatin structure; and alanine released from stromal cells sustains mitochondrial metabolism and therapy resistance. These dependencies are closely tied to amino acid transporters, which act as both nutrient entry points and measurable biomarkers. In this review, we summarize current evidence on histidine, isoleucine, threonine, lysine, and alanine as emerging metabolic targets, and discuss opportunities and challenges for clinical translation, with emphasis on transporter biology, biomarker development, and therapeutic combinations.

## 1 Introduction

Amino acids play a central role in cancer metabolism, serving as building blocks for protein synthesis and regulators of signaling, redox balance, and immune function (Liu et al., 2024). They are often classified as essential, non-essential, or conditionally essential, and further grouped by structural or metabolic features such as branched-chain amino acids (BCAAs: leucine, isoleucine, valine), aromatic amino acids (tryptophan, phenylalanine, tyrosine), and sulfur-containing amino acids (methionine, cysteine). These categories are associated with distinct biological roles in cancer research. For example, BCAAs activate mechanistic target of rapamycin complex 1 (mTORC1), a growth pathway that senses nutrient availability (Saxton and Sabatini, 2017). Serine, glycine, and methionine feed one-carbon (1C) metabolism, which provides nucleotides for DNA synthesis and supports epigenetic regulation (Ducker and Rabinowitz, 2017), while arginine and tryptophan regulate immune responses via T-cell activity and kynurenine–aryl hydrocarbon receptor (AhR) signaling (Carpentier et al., 2024; Ghorani et al., 2023).

Disruptions in amino acid pathways are a hallmark of cancer. Cancer cells reprogram amino acid uptake and utilization to sustain biomass accumulation, maintain antioxidant defenses, and promote growth signaling (Saxton and Sabatini, 2017). These adaptations create differences in tumor and normal tissues that can be therapeutically exploited. Clinical studies have already established proof-of-concept. For example, arginine depletion has shown activity in tumors deficient in argininosuccinate synthase 1 (ASS1) (Chan et al., 2021; Chan et al., 2022; Szlosarek et al., 2021), the enzyme that enables cells to synthesize arginine *de novo*. Glutaminase inhibitors, which block the enzyme responsible for converting glutamine into glutamate and feeding the tricarboxylic acid (TCA) cycle, have been evaluated in glutamine-dependent cancers, both as single drug therapy and in rational drug combinations (Patel et al., 2024; Wicker et al., 2021; DiNardo et al., 2024). Similarly, inhibition of tryptophan catabolism through indoleamine 2,3-dioxygenase (IDO) and tryptophan 2,3-dioxygenase (TDO), enzymes that degrade tryptophan to kynurenine, has been explored to restore antitumor immunity (Wu et al., 2023; Zakharia et al., 2021). These studies establish the feasibility of targeting amino acid metabolism in patients and demonstrate two recurring challenges: tumors can rewire their metabolism in ways that allow them to escape treatment, and the lack of reliable biomarkers makes it difficult to identify which patients are most likely to benefit from treatment (Akinlalu et al., 2025; Balamurugan et al., 2025; Rasuleva et al., 2023; Rasuleva et al., 2021). Recent advances have begun to address these limitations, with ASS1 loss used as a biomarker for arginine deprivation (Carpentier et al., 2024), kynurenine-to-tryptophan ratios to monitor IDO/TDO inhibition (Wu et al., 2023; Huang et al., 2022), and circulating metabolites or amino acid-based positron emission tomography (PET) tracers to confirm drug activity (Galldiks et al., 2023). These developments represent important steps toward more precise, biomarker-guided use of amino acid therapies.

In parallel, new amino acid targets have come into focus. In pancreatic cancer, histidine and isoleucine supplementation are selectively cytotoxic to cancer cells while sparing non-malignant counterparts (Akinlalu et al., 2024). In glioblastoma (GBM), threonine drives tumor growth by fueling codon-biased protein synthesis through transfer RNA (tRNA) modifications mediated by the enzyme YRDC (Wu et al., 2024). Lysine catabolism promotes immune evasion by altering histone modifications (crotonylation) that suppress interferon signaling (Yuan et al., 2023). Alanine released by stromal fibroblasts fuels mitochondrial metabolism in cancer cells, enabling survival and resistance to therapy (Zhu et al., 2023; Gauthier-Coles et al., 2022).

Importantly, these emerging amino acid targets are linked to their associated transporters that act as nutrient gateways and functional biomarkers. The L-type amino acid transporter 1 (LAT1; gene name *SLC7A5*) mediates uptake of histidine, isoleucine, and threonine (Bo et al., 2021). The cationic amino acid transporter 1 (CAT1; gene name *SLC7A1*) transports lysine and contributes to immune regulation (You et al., 2022). The sodium-coupled neutral amino acid transporter 2 (SNAT2; gene name *SLC38A2*) facilitates the uptake of alanine (Gauthier-Coles et al., 2022). Advances in transporter inhibitors (Bo et al., 2021; Lyu et al., 2023), metabolic imaging using amino acid PET tracers (Galldiks et al., 2023; Jakobsen et al., 2023), and studies linking transporter activity to immune regulation (Bo et al., 2021; Guo et al., 2023) underscore their central role in clinical translation.

**FIGURE 1:**
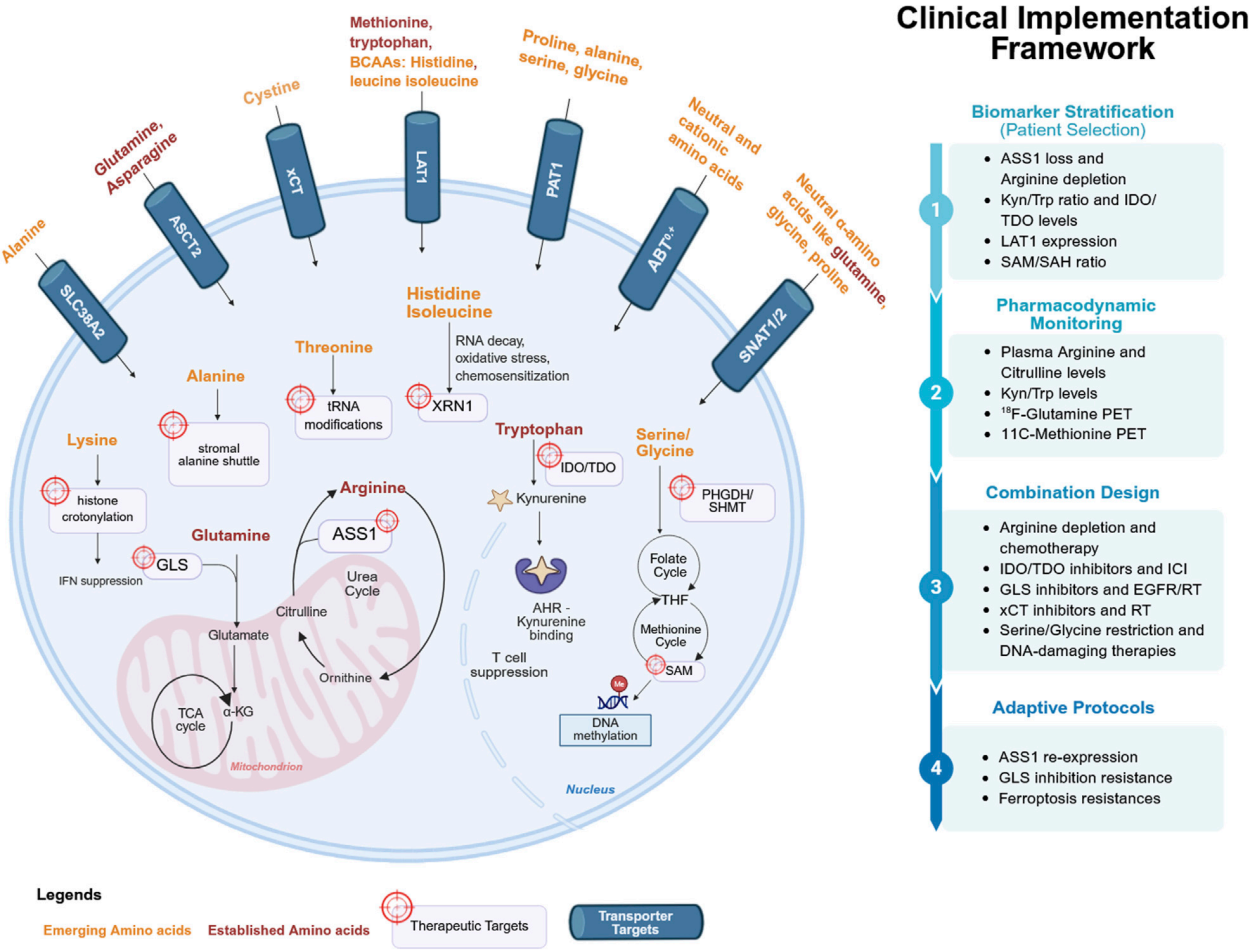
Amino acid metabolism and transporters as therapeutic targets in cancer. The cellular pathway illustrates the tumor cell with established dependencies (arginine, glutamine, tryptophan, methionine, asparagine; red) and emerging amino acids (histidine, proline, serine–glycine, aspartate, branched-chain amino acids, threonine, lysine, alanine; orange). Transporters (LAT1, ASCT2, xCT, SLC6A14, SNAT1/2, PAT1; blue) are localized at the plasma membrane. Arrows represent nutrient influx and metabolic pathways, with icons denoting therapeutic interventions (enzyme depletion, pharmacologic inhibitors, dietary restriction, transporter blockade). The clinical implementation framework emphasizes biomarker stratification, pharmacodynamic monitoring, combination design, and adaptive protocols, reflecting how amino acid biology is translated into precision oncology. Figure was created in Biorender. Akinlalu, A. (2025).

Here, we focus on histidine, isoleucine, threonine, lysine, and alanine as emerging metabolic vulnerabilities in cancer. We emphasize their mechanism, associated transporters, and translational relevance. Drawing on lessons from established amino acid interventions, we outline the opportunities and challenges that will shape the clinical development of these emerging strategies (Figure 1; Table 1).

**TABLE 1:** Established and emerging amino acid dependencies and transporter targets in cancer. The table summarizes mechanisms, transporters, biomarkers, strategies, and evidence tiers for the four established pathways (arginine, glutamine, tryptophan, methionine) and five emerging amino acid cancer therapy, histidine, isoleucine, threonine, lysine and alanine. Transporters central to these targets are shown as both therapeutic entry points and functional biomarkers.

Target/Transporter	Therapeutic strategy	Cancer type(s)	Mechanism of action	Biomarker(s)	Clinical stage	References
Arginine/ASS1	Pegargiminase (ADI-PEG 20), rhArg, combinations with chemotherapy and ICI	Mesothelioma, HCC, NSCLC, Sarcoma	Arginine depletion; mTORC1 and polyamine suppression	ASS1 loss (IHC, methylation), plasma arginine/citrulline	Phase II/III	Carpentier et al. (2024), Chan et al. (2021), Chan et al. (2022), Szlosarek et al. (2021), Szlosarek et al. (2024), Rogers et al. (2023), Strømland et al. (2025)
Tryptophan/IDO1-TDO2-AHR	IDO1/TDO2 inhibitors, AHR antagonists, and PD-1 blockade	Melanoma, NSCLC, Colon	Kynurenine accumulation, AHR-mediated immunosuppresion	Kyn/Trp ratio, IDO/TDO expression, AHR activity	Phase I/II	(Wu et al., 2023; Tokito et al., 2024; Yap et al., 2025; Schlichtner et al., 2023; Campesato et al., 2020)
Glutamine/Glutaminase	GLS inhibitors (CB-839), diet and targeted therapy	Renal, Colon, TNBC	Blockade of glutamine anaplerosis; impaired nucleotide synthesis, redox stress	GLS expression, LAT1/ASCT 2 profile,^18^F-Gln PET	Phase I/II	(Patel et al., 2024; Wicker et al., 2021; DiNardo et al., 2024; Ciombor et al.,2025; Tsujimoto et al., 2023; Chen et al., 2025; Gouda et al., 2025; Kuroki et al., 2023; Lee et al., 2022)
Methionine/One-carbon	Dietary restriction, methioninase, MAT2A/PHGDH/SHMT inhibitors	Prostate, Colon, Pancreas, glioma	SAM depletion; epigenetic deregulation, impaired 1C flux	SAM/SAH ratios, PHGDH/SHMT expression	Phase I/Preclinical	(Mattes et al., 2024; Han and Hoffman, 2021; Bernasocchi and Mostoslavsky, 2024; Tong et al., 2024; Gounder et al., 2025)
Histidine	Dietary supplementation and chemo/radiotherapy	Pancreas	Redox stress via GSH depletion/ROS; RNA turnover stress	Low plasma histidine; ROS/GSH	Preclinical	(Akinlalu et al., 2024; Kumar et al., 2023)
Isoleucine and BCAAs	Acute/pulsed supplementation	PDAC	mTORC1 activation, EV-mediated amino acid overload	BCAA plasma levels; stress markers	Preclinical	(Akinlalu et al., 2024; Matsui et al., 2024; Ma et al., 2024)
Threonine	Dietary restriction; YRDC/tRNA modification pathway inhibition	Glioblastoma	tRNA modification codon-biased translation and proliferation	YRDC, tRNA-modification enzymes accumulation	Preclinical	(Wu et al., 2024; Harris et al., 2011)
Lysine	Dietary restriction; inhibit lysine catabolism	Glioblastoma; HCC	Catabolism, crotonyl-CoA, histone crotonylation, IFN suppression	Crotonylation markers; GCDH elevation, IFN signatures	Preclinical	(Yuan et al., 2023; Jing et al., 2025; Johal et al., 2024)
Alanine/SNAT2	Block SNAT2 uptake or alanine overload	PDAC, ovarian, SMARCA4/2-deficient tumors	Stromal alanine shuttle fueling anaplerosis; genotype-selective dependence	SNAT2 and GPT2 elevation; stromal-tumor alanine flux	Preclinical	(Zhu et al., 2023; Nie et al., 2025)
LAT1 (*SLC7A5*)	JPH203 transporter inhibition, imaging-guided targeting	Glioma, NSCLC, breast, GI	Leu/Ile/Met uptake, mTORC1 activation	LAT1 expression,^11^C-Met PET	Early Clinical	(Galldiks et al., 2023; Bo et al., 2021; Sato et al., 2021)
ASCT2 (*SLC1A5*)	C118P, siRNA, small-molecule inhibitors	Breast, PDAC	Glutamine uptake, supports glutamine addiction	ASCT2 expression	Preclinical	(Lyu et al., 2023; Shi et al., 2024)
xCT (*SLC7A11*)	Sulfasalazine, sorafenib, ferroptosis sensitizers	Colon, liver, lung, pancreas	Inhibition depletes glutathione and increases ferroptotic susceptibility	SLC7A11 expression, GSH/GSSG ratios	Early clinical/Preclinical	(Hu et al., 2020; He et al., 2025; Yan et al., 2023; Messier et al., 2024)
SNAT1/2 (*SLC38A1/2*)	Transporter inhibition	Breast, ovarian	Alanine uptake; stress-induced upregulation	SLC38A1/2 expression	Preclinical	(Jakobsen et al., 2023; Gauthier-Coles et al., 2022)

## 2 Emerging amino acids in cancer therapy

### 2.1 Histidine

Histidine is an essential amino acid with unique chemical properties. Its imidazole side chain can buffer protons and bind metals, giving histidine a central role in pH balance and metal-dependent enzymatic reactions (Kessler and Raja, 2023). Historically, histidine has not been considered a major driver of cancer metabolism. However, recent work in pancreatic ductal adenocarcinoma (PDAC) has highlighted histidine as a potential therapeutic target.

Our recent study (Akinlalu et al., 2024) demonstrated that histidine combined with isoleucine induces selective cytotoxicity in PDAC. Across *in vitro* cell culture models and *in vivo* nude mice xenografts, supplementation with these two amino acids reduced PDAC cell viability while sparing non-malignant counterparts. This selectivity suggests an intrinsic sensitivity to histidine-isoleucine overload, likely linked to altered amino acid handling by tumor cells. The study also pointed to the mRNA exonuclease XRN1 as a possible mechanism: when histidine and isoleucine accumulated, RNA processing was perturbed, adding to cellular stress. Rather than starving tumors of nutrients, this approach overloads them with amino acids they cannot manage, leading to metabolic crisis and cell death.

A complementary study (Kumar et al., 2023) independently showed that histidine supplementation disrupts tumor metabolic balance through a different mechanism. In PDAC models, histidine overload depleted amino acids required for glutathione synthesis, leading to loss of redox homeostasis, accumulation of hydrogen peroxide, and oxidative stress. This sensitized tumors to gemcitabine; exogenous glutathione rescued the effect, confirming the oxidant-antioxidant mechanism. These data suggest two converging mechanisms in PDAC: interference with RNA processing when histidine is paired with isoleucine, and induction of oxidative stress when histidine is used alone. Both selectively stress PDAC cells over normal tissues. While these preclinical findings are compelling, their current limitation lies in model specificity, as most evidence arises from xenograft and cell-line systems. Independent confirmation in patient-derived models and pharmacokinetic studies will be necessary to determine whether histidine modulation is feasible and safe in clinical settings.

Translationally, histidine could be explored as an adjuvant to chemotherapy, as a co-supplement with isoleucine to induce direct cytotoxicity, or in combination with therapies that generate oxidative stress. Recent studies show that PDAC tumors exhibit abnormal histidine uptake and catabolism mediated by histidine ammonia-lyase (HAL), which may contribute to reduced circulating histidine levels and increased oxidative stress (Kumar et al., 2023; Wu et al., 2025; McDonnell et al., 2025). Although stromal barriers in PDAC may limit direct correspondence between plasma and intratumoral metabolites, these observations suggest that HAL expression or activity within the tumor, and possibly systemic histidine availability, may serve as exploratory biomarkers for identifying patients most likely to benefit from histidine-based interventions.

### 2.2 Isoleucine

Isoleucine is one of the BCAAs, along with leucine and valine. BCAAs support protein synthesis, activate mTORC1, and influence immune function (Dimou et al., 2022). In PDAC cells, proteomic analysis of extracellular vesicles (EVs) suggests that tumor cells actively dispose of isoleucine and histidine, consistent with intracellular accumulation being stressful (Akinlalu et al., 2024). Re-supplementation of isoleucine, alone or with histidine, overwhelmed PDAC cells and triggered necrotic death, while non-malignant cells tolerated supplementation (Akinlalu et al., 2024).

Clinically, isoleucine supplementation may be feasible because BCAA-enriched nutrition is used in perioperative and supportive care for cancer patients. Meta-analyses and randomized trials in gastrointestinal cancers report improved nitrogen balance, fewer infections, and better postoperative recovery with BCAAs (Matsui et al., 2024; Ma et al., 2024). This safety profile suggests that controlled isoleucine supplementation could be integrated into therapeutic regimens. However, dosing will require careful monitoring to avoid systemic imbalance. While short pulses of high isoleucine may selectively stress tumors, prolonged elevation of circulating BCAAs could increase the risk of side effects, such as insulin resistance or neurological complications (Shah et al., 2024; De Simone et al., 2013).

### 2.3 Threonine

Threonine is essential for protein synthesis and one-carbon metabolism (Tang et al., 2021). In GBM, one of the most aggressive brain tumors, threonine plays a distinct role in translational control, growth and survival (Wu et al., 2024). Wu et al. (2024) demonstrated that GBM cells accumulate unusually high levels of threonine, which fuels a specific tRNA modification (Wu et al., 2024). The enzyme YRDC (YrdC domain–containing protein) uses threonine to generate N^6^-threonylcarbamoyl adenosine (t^6^A), a modification found on tRNAs that decode ANN codons (Perrochia et al., 2013; Harris et al., 2011). This modification skews protein translation toward mitosis-related and proliferative proteins, giving GBM cells a growth advantage. When threonine availability was reduced or YRDC was inhibited, GBM cells showed impaired t^6^A modification, reduced protein synthesis, and suppressed proliferation. In mouse models, dietary threonine restriction significantly slowed tumor growth, validating threonine as a nutrient that sustains GBM progression (Wu et al., 2024).

For clinical translation, two approaches may be explored. First, dietary modulation, where controlled threonine restriction could selectively stress tumors with high translational demand while sparing normal tissues. Second, pharmacological inhibition of YRDC or related enzymes in the tRNA modification pathway. Such drugs could directly impair the codon-biased translation mechanism that GBM depends on. Although this modification has been observed in a subset of GBM models, potential biomarkers may include YRDC enzymatic activity, high t^6^A modification levels in tumor RNA, or metabolic features of threonine accumulation. Because these findings are still limited, larger patient studies will be needed to validate these biomarkers and to determine which tumors are most likely to respond to threonine-targeted therapy. Moreover, while threonine availability has been mechanistically linked to YRDC-dependent translation and tumor growth, this evidence comes from GBM models and tumor subtypes. Whether similar vulnerabilities occur across other tumor types or in patients remains uncertain, demonstrating the need for broader validation and biomarker-guided trial designs.

### 2.4 Lysine

Lysine is an essential amino acid that serves as both a protein building block and a site for multiple post-translational modifications, including acetylation, ubiquitination, and methylation (Wang and Cole, 2020). In GBM, excess lysine breakdown produces crotonyl-CoA, which drives histone crotonylation and suppresses type I interferon signaling (Yuan et al., 2023). This epigenetic silencing weakens immune surveillance by reducing cytotoxic T cell infiltration into tumors. Limiting lysine availability, through dietary restriction or inhibition of catabolic enzymes, lowers crotonyl-CoA levels, restores interferon pathways, and enhances the response to immunotherapy. These findings place lysine metabolism at the center of a metabolic–epigenetic control that tumors exploit to hide from the immune system.

Also, lysine modulation can act in multiple directions depending on tumor type and context. In another GBM study, restricting lysine or using lysine mimics that compete with its metabolic functions enhanced the efficacy of standard chemotherapy, demonstrating the value of nutrient limitation approaches (Jing et al., 2025). Conversely, in hepatocellular carcinoma (HCC), lysine transporter-mediated uptake improved the effects of targeted therapy combined with immune checkpoint inhibition (ICI), indicating that in some tumors, additional lysine may tip the balance toward improved immune activity (Chang et al., 2025). Meanwhile, systemic studies in mice have shown that lysine restriction is physiologically tolerable and can reduce the buildup of harmful lysine catabolites, further supporting the safety of metabolic modulation (Johal et al., 2024).

These results illustrate that lysine-based therapies cannot be viewed through a single lens of “restriction” or “supplementation”. Instead, lysine represents a flexible metabolite whose impact depends on the interplay between tumor metabolism, the immune microenvironment, and therapy context. Other lysine-derived modifications, including succinylation and lactylation, are gaining attention as regulators of gene expression and immune signaling, pointing to additional therapeutic targets in lysine metabolism.

For clinical translation, lysine restriction may be advantageous in tumors that exploit catabolism to evade immunity, while supplementation could enhance therapy in settings where immune cells compete with tumors for lysine. Future clinical translation will require careful patient selection, biomarker development, and context-specific strategies that account for this duality.

### 2.5 Alanine

Alanine, a non-essential amino acid, is increasingly recognized as a fuel in the metabolic cooperation between tumor cells and their microenvironment. Beyond its traditional role in the glucose-alanine cycle between muscle and liver, alanine has been shown to act as a stromal-derived nutrient that sustains tumor mitochondrial metabolism (Parker et al., 2020). In PDAC, pancreatic stellate cells release alanine into the tumor microenvironment, which is subsequently imported by cancer cells and converted to pyruvate (Be et al., 2019). Pyruvate replenishes the TCA cycle through anaplerosis, the process of refilling depleted metabolic intermediates. This exchange allows PDAC cells to preserve oxidative phosphorylation and biosynthetic activity under nutrient stress, demonstrating alanine as a central metabolite in stromal-tumor metabolic crosstalk.

Alanine dependence has also been observed in other cancers with defined genetic backgrounds. In ARID1A-mutant ovarian cancers, where loss of the ARID1A chromatin-remodeling gene rewires metabolic programs, tumor cells show heightened reliance on alanine uptake (Nie et al., 2025). Blocking this uptake impaired tumor growth in preclinical models, revealing a genotype-selective vulnerability. Similarly, in SMARCA4/2-deficient tumors, which lack components of the SWI/SNF chromatin-remodeling complex, exogenous alanine overload demonstrated cytotoxicity (Zhu et al., 2023). Supplementation with alanine disrupted metabolic balance and induced cell death in cell cultures and xenograft models (Zhu et al., 2023). These findings suggest that the role of alanine in cancer is not uniform but shaped by tumor genotype, with certain mutations conferring heightened susceptibility. A notable strength of these studies is the integration of metabolic tracing and co-culture systems, which reveal real-time nutrient exchange between stromal and cancer cells. However, translating these observations remains challenging, as stromal heterogeneity and tissue architecture may alter alanine dynamics *in vivo*.

Clinically, targeting alanine metabolism presents dual opportunities. One approach is to inhibit alanine uptake by blocking sodium-coupled neutral amino acid transporter 2 (SNAT2; *SLC38A2*), which mediates alanine import. Early studies indicate that pharmacological inhibition of SNAT2 suppresses tumor growth and can synergize with inhibitors of glucose metabolism (Gauthier-Coles et al., 2022). Alternatively, in specific genetic contexts such as SMARCA4/2 deficiency, alanine supplementation itself may be leveraged to overwhelm tumor metabolic capacity, a strategy that contrasts with nutrient-depletion approaches.

## 3 Amino acid transporters as targets and functional biomarkers

### 3.1 LAT1 (*SLC7A5*)

L-type amino acid transporter 1 (LAT1) mediates the uptake of large neutral amino acids such as, leucine, and threonine (Scalise et al., 2018a). LAT1 is frequently overexpressed in solid tumors, including pancreatic, breast, and brain cancers (Sato et al., 2021; Okano et al., 2020). High LAT1 expression correlates with poor prognosis and resistance to standard therapies, reflecting its role in fueling mTORC1 signaling (Shibasaki et al., 2023). Pharmacological inhibitors of LAT1, such as JPH203, have entered early-phase clinical testing and demonstrated manageable safety profiles with preliminary signs of antitumor activity (Bo et al., 2021; Okano et al., 2020). More recent studies suggest that LAT1 also modulates the tumor immune microenvironment by controlling the amino acid supply to both tumor cells and infiltrating lymphocytes, making it a potential dual-purpose target (Zhao et al., 2025).

### 3.2 ASCT2 (*SLC1A5*)

The alanine/serine/cysteine transporter 2 (ASCT2; gene name *SLC1A5*) primarily mediates glutamine uptake but also transports neutral amino acids. While glutamine metabolism is well established in cancer, ASCT2 is a vital target in nutrient uptake. Inhibitors such as C118P block ASCT2-mediated transport and demonstrate antitumor efficacy in preclinical breast cancer models (Lyu et al., 2023). Recent structural studies have improved the understanding of ASCT2’s substrate-binding dynamics, allowing for new inhibitors with greater selectivity. Because ASCT2 expression can be detected via immunohistochemistry or transcriptomic profiling, it is also being evaluated as a biomarker for glutamine dependence in patient tumors (Lyu et al., 2023; Garibsingh et al., 2021).

### 3.3 xCT (*SLC7A11*)

The cystine/glutamate antiporter (xCT; gene name *SLC7A11*) imports cystine in exchange for glutamate, coupling amino acid transport to redox homeostasis through glutathione synthesis (Hu et al., 2020). Overexpression of xCT protects tumors from oxidative stress but also creates therapeutic opportunities: targeting xCT sensitizes cancers to radiotherapy and ferroptosis-inducing agents (He et al., 2025; Yan et al., 2023). Recent work has shown that xCT levels predict response to radiotherapy in colorectal cancer liver metastases, highlighting its value as both a target and a clinical biomarker (He et al., 2025). Additionally, inhibitors of xCT are being explored in combination with immune checkpoint inhibitors, where depletion of cystine may amplify T cell–mediated oxidative killing (He et al., 2025; Messier et al., 2024; Xu et al., 2021).

### 3.4 SNAT2 (*SLC38A2*)

The sodium-coupled neutral amino acid transporter 2 (SNAT2; gene name *SLC38A2*) regulates transport of alanine and other small neutral amino acids. SNAT2 expression is upregulated in nutrient-stressed tumors and correlates with therapy resistance (Gauthier-Coles et al., 2022). In ovarian cancers with ARID1A mutations, reliance on SNAT2-mediated alanine uptake has been identified as a selective vulnerability (Gauthier-Coles et al., 2022). Pharmacological inhibitors of SNAT2 are in preclinical development and demonstrate synergy with glucose metabolism inhibitors, demonstrating their role as a metabolic checkpoint (Jakobsen et al., 2023; Gauthier-Coles et al., 2022).

### 3.5 CAT1 (*SLC7A1*)

The cationic amino acid transporter 1 (CAT1; gene name *SLC7A1*) imports positively charged amino acids such as lysine and arginine. Recent studies link CAT1 to immune regulation, showing that lysine transport influences epigenetic programs that modulate interferon responses (You et al., 2022). In GBM, upregulation of lysine transport enhances histone crotonylation, leading to suppression of type I interferon signaling and immune evasion (Yuan et al., 2023). Although CAT1 has not yet been the focus of clinical trials, its role in metabolic–epigenetic reprogramming highlights its translational potential.

### 3.6 Transporters as imaging and circulating biomarkers

Amino acid transporters also provide opportunities for non-invasive imaging and biomarker development. Radiolabeled amino acid analogs such as ^18^F-fluoroethyltyrosine (FET) and ^11^C-methionine are used in PET to visualize transporter activity *in vivo* (Galldiks et al., 2023). Recent studies explore the use of LAT1-specific tracers to monitor tumor metabolism and treatment response dynamically (Achmad et al., 2025). In parallel, circulating metabolite profiles, such as plasma amino acid ratios, are being tested as functional markers of transporter activity, offering accessible biomarkers for patient selection in clinical trials (Chan et al., 2021; Zakharia et al., 2021; Chang et al., 2025).

### 3.7 Clinical and safety considerations for amino acid transporter-targeted therapies

As amino acid transporters continue to emerge as therapeutic targets, it is important to consider their functions in normal physiology to ensure treatment safety. Most of these transporters are not tumor-specific; they are also expressed in healthy tissues that rely on continuous amino acid exchange. LAT1, for example, is highly active at the blood–brain barrier and placenta, mediating the transfer of large neutral amino acids essential for brain and fetal development (Ohgaki et al., 2017). ASCT2 and SNAT1/2 regulate the movement of neutral amino acids across liver, muscle, and kidney cells, contributing to energy metabolism and osmotic balance (Scalise et al., 2018b; Ge et al., 2018), while xCT maintains antioxidant defense by exchanging cystine and glutamate in many epithelial tissues (Martis et al., 2020). Because of these physiological roles, broad or prolonged inhibition of these transporters can potentially cause nutrient imbalance, neurotoxicity, or organ dysfunction if monitoring is inadequate or drug exposure is not properly controlled. Early clinical and preclinical studies indicate that transporter inhibitors can be administered safely when dosing is well managed, but they also highlight the need for continuous safety assessment given the essential metabolic functions of these proteins. Emerging strategies such as tumor-targeted delivery systems, intermittent or adaptive dosing, and combination therapies are being developed to minimize systemic exposure while maintaining anti-tumor activity. Although transporter-targeting strategies such as LAT1 and ASCT2 inhibition show reproducible metabolic effects, clinical translation is constrained by transporter redundancy and expression in normal organs. These factors complicate dose optimization and emphasize the need for transporter-selective or tumor-targeted approaches. Integrating transporter biology with pharmacokinetic monitoring in future trials will be crucial for translating these approaches safely into clinical use.

## 4 Oncogenic regulation, opportunities and challenges for clinical translation

### 4.1 Oncogenic regulation of amino acid metabolism and transporters

Amino acid metabolism and transporter activity are tightly linked to oncogenic signaling. Many of the enzymes and transporters discussed in this review are directly regulated by transcription factors that coordinate nutrient use with cell growth and stress response. MYC (myelocytomatosis oncogene), one of the most studied oncogenes, increases the expression of several amino acid transporters, including ASCT2 and LAT1, to enhance amino acid uptake and sustain mTORC1 activity (Hansen et al., 2015). YAP (Yes-associated protein) and TAZ (transcriptional coactivator with PDZ-binding motif), the downstream effectors of the Hippo pathway, also stimulate amino acid transport by upregulating LAT1, which promotes leucine uptake and mTORC1 signaling, especially under nutrient-limited conditions (Hansen et al., 2015; Bertolio et al., 2023). In addition, mutant p53 (tumor protein p53) reprograms amino acid metabolism by promoting serine–glycine synthesis and LAT1/CD98 expression, while repressing xCT through interaction with NRF2 (nuclear related factor 2), thereby altering redox balance and nutrient handling (Tombari et al., 2023). These oncogenic influences show that metabolic rewiring is not a passive adaptation to nutrient stress but a genetically driven feature of tumor progression. Recognizing how oncogenic drivers shape amino acid dependencies can guide both biomarker development and inform the design of pathway-specific therapeutic combinations.

### 4.2 Opportunities for translational targeting

#### 4.2.1 Biomarker-driven patient selection

Clinical experience with arginine and tryptophan has shown that amino acid metabolism is most effectively targeted when linked to biomarkers, such as ASS1 loss for arginine or the kynurenine/tryptophan ratio for tryptophan catabolism. For emerging amino acids, parallel opportunities exist: plasma histidine depletion or high expression of histidine ammonia-lyase (HAL) may predict response to histidine–isoleucine supplementation; YRDC catalytic activity or elevated tRNA modification signatures could identify threonine-dependent glioblastomas; lysine-crotonylation signatures or GCDH overexpression may stratify patients for lysine restriction; and transporter expression, such as SNAT2 for alanine or LAT1 for histidine/isoleucine/threonine, could provide functional markers of dependency.

#### 4.2.2 Exploiting nutrient surplus as a therapeutic target

Most metabolic therapies have focused on nutrient deprivation, such as starving tumors of arginine or methionine. By contrast, histidine and isoleucine supplementation demonstrate that amino acid supplementation can selectively induce tumor stress. This conceptual shift broadens the therapeutic approach, allowing tumors’ own metabolic vulnerabilities (such as amino acid export or catabolic upregulation) to be used against them.

#### 4.2.3 Combination therapies and synergy

Just as glutaminase inhibition has shown greatest promise in combination with radiation or chemotherapy, emerging amino acid strategies may synergize with existing treatments. Histidine supplementation enhances gemcitabine activity by disrupting redox balance, lysine restriction boosts immune checkpoint blockade by reactivating interferon pathways, and alanine modulation sensitizes tumors to mitochondrial stress and chemotherapy. Rationally designed combinations could overcome the limited durability seen with single therapies.

#### 4.2.4 Transporters as tractable drug targets and imaging tools

LAT1, SNAT2, and CAT1 represent pharmacologically accessible points that couple extracellular nutrient supply with tumor growth. Notably, transporter activity can also be visualized non-invasively through PET tracers, enabling real-time monitoring. This dual role, both as targets and biomarkers, strengthens the translational case for amino acid–based therapies.

### 4.3 Challenges

#### 4.3.1 Tumor microenvironment metabolism and therapy resistance

Amino acid metabolism in tumors represents a network shared between cancer cells, stromal fibroblasts, and immune populations within the tumor microenvironment (TME). This metabolic cooperation shapes both nutrient access and treatment response. Cancer-associated fibroblasts (CAFs) can secrete alanine, sustaining oxidative metabolism in pancreatic tumors during nutrient stress (Sousa et al., 2016), while aspartate-glutamate exchange between stromal and tumor cells buffers redox status and supports biosynthesis (Be et al., 2019). Within the immune compartment, depletion of tryptophan by IDO1/TDO2 and arginine by myeloid cells suppresses T-cell proliferation and effector function (Günther et al., 2019). Beyond these established pathways, emerging amino acid dependencies, such as those involving histidine, isoleucine, threonine, lysine, or alanine, may further influence the TME by modifying oxidative balance, nutrient signaling, and stress adaptation. Integrating these newer observations with known mechanisms suggests that tumors exploit a flexible amino acid economy in which both stromal support and immune suppression converge to sustain growth. This metabolic reciprocity explains why amino acid-targeted interventions often show context-dependent efficacy and demonstrates the need for therapeutic strategies that account for tumor microenvironmental nutrient exchange, immune cell competition, and tumor metabolic plasticity. These tumor-stromal-immune exchanges illustrate the multifactorial nature of amino acid metabolism in the TME, setting the stage for broader challenges in clinical translation. Collectively, the studies reviewed provide valuable mechanistic insight but vary in translational depth. Many rely on metabolic or xenograft models that simplify the TME, whereas patient-derived organoids and isotope tracing in clinical samples remain limited. These methodological differences partly explain why results are sometimes inconsistent across tumor types. Recognizing these constraints allows a more accurate assessment of which amino-acid pathways are most actionable in patients.

#### 4.3.2 Metabolic plasticity and adaptive resistance

Experience with arginine and glutamine has shown how tumors adapt when a single nutrient pathway is blocked, rerouting flux through compensatory pathways. Emerging strategies will face similar resistance: tumors may adjust transporter expression, shift metabolic routing, or draw on stromal support to bypass amino acid targeting.

#### 4.3.3 Balancing systemic safety with tumor selectivity

Unlike small-molecule inhibitors with defined targets, dietary or metabolic interventions risk perturbing systemic amino acid pools. Although preclinical studies suggest that histidine, isoleucine, and threonine interventions are tolerated, long-term effects on protein synthesis, immune function, and normal tissue homeostasis must be carefully evaluated.

#### 4.3.4 Heterogeneity of tumor microenvironment

Alanine exemplifies how stromal fibroblasts supply tumors with metabolic support. This highlights a challenge: vulnerabilities may not arise from tumor cells alone but from the interaction between tumor and microenvironment. Therapies will need to consider this complexity, as targeting stromal-tumor nutrient exchange is inherently more difficult than inhibiting tumor-intrinsic enzymes.

#### 4.3.5 Limited clinical data and trial design hurdles

While arginine and glutamine have advanced into randomized trials, most emerging amino acid strategies remain in early preclinical stages. Translating them will require innovative trial designs, such as adaptive protocols that pivot therapy upon biomarker-detected resistance, as well as careful patient selection to ensure only biomarker-positive patients are enrolled.

### 4.4 Future direction

The successful translation of emerging amino acid therapies will depend on a precision-guided approach. This means linking basic mechanistic insights with reliable biomarkers, designing smart treatment combinations, and selecting patients based on their genetic or metabolic profile. For example, methionine restriction is being tested with radiation therapy, and arginine depletion is used in patients whose tumors lack ASS1. In the same way, new amino acid strategies must align the biology of each target with matching biomarkers and treatment designs. Equally important, future progress will require integrating these mechanistic findings with carefully designed clinical studies that address current limitations in model relevance, patient selection, and biomarker validation. Combining metabolic profiling with functional assays in patient-derived systems will help distinguish true causal dependencies from correlative observations, ensuring that only the most actionable pathways advance into clinical testing. Incorporating transporter biology, monitoring plasma metabolite levels, and building rational combinations with existing therapies will be key to moving these approaches from preclinical promise to real clinical benefit.

## 5 Conclusion

Amino acid metabolism is a promising frontier in cancer research. Established interventions such as arginine depletion, glutaminase inhibition, tryptophan catabolism blockade, and methionine restriction have demonstrated both the feasibility and the complexity of targeting metabolic pathways in patients. Building on these lessons, recent discoveries highlight histidine, isoleucine, threonine, lysine, and alanine as new amino acid dependencies with translational potential. Each of these amino acids introduces distinct mechanisms, from selective cytotoxicity and redox imbalance to translational control, epigenetic reprogramming, and stromal–tumor nutrient exchange. Notably, these new targets are closely tied to amino acid transporters such as LAT1, CAT1, and SNAT2, which function as metabolic gateways and potential therapeutic targets or imaging biomarkers. This dual role strengthens the rationale for incorporating transporters into biomarker-driven clinical strategies. Opportunities include exploiting nutrient surplus, pairing amino-acid modulation with chemotherapy, radiotherapy, and immunotherapy, and deploying functional biomarkers for patient selection. Challenges include metabolic plasticity, microenvironmental heterogeneity, and the need for careful trial design to balance systemic safety with tumor selectivity. Looking forward, progress will depend on precision-oriented implementation that combines mechanistic insight with biomarker development and rational therapeutic combinations. By aligning amino acid mechanisms with transporter targeting, metabolic imaging, and patient selection, the field can move closer to translating emerging amino acid dependencies into durable clinical benefit. Ultimately, integrating these strategies into precision oncology frameworks has the potential to expand treatment options and improve outcomes for patients with some of the most difficult-to-treat cancers.

## Glossary

1C: One carbon
AA: Amino acid
ADI-PEG 20: Pegylated arginine deiminase
AHR: Aryl Hydrocarbon Receptor
ALT2/GPT2: Alanine Aminotransferase 2 (Glutamic-Pyruvic Transaminase 2)
ASCT2 (*SLC1A5*): Alanine-Serine-Cysteine Transporter 2
ASS1: Argininosuccinate Synthase 1
BCAAs: Branched-Chain Amino Acids
CAT1 (*SLC7A1*): Cationic amino acid transporter 1
EVs: Extracellular vesicles
GBM: Glioblastoma (glioblastoma multiforme)
GCDH: Glutaryl-CoA dehydrogenase
GLS: Glutaminase
GSH: Reduced glutathione (GSSG = oxidized glutathione)
HCC: Hepatocellular carcinoma
HAL: Histidine ammonia-lyase
ICI: Immune checkpoint inhibitor
IDO1/TDO2: Indoleamine 2,3-dioxygenase 1/Tryptophan 2,3-dioxygenase 2
IHC: Immunohistochemistry
Kyn/Trp: Kynurenine-to-tryptophan ratio
LAT1 (*SLC7A5*): L-type amino acid transporter 1
mTORC1: Mechanistic target of rapamycin complex 1
NSCLC: Non-small cell lung cancer
PD-1: Programmed cell death protein 1
PDAC: Pancreatic ductal adenocarcinoma
PET: Positron emission tomography
ROS: Reactive oxygen species
SLC: Solute carrier (transporter family prefix)
SNAT2 (*SLC38A2*): Sodium-coupled neutral amino acid transporter 2
TCA cycle: Tricarboxylic acid cycle
**t** ^**6**^ **A**: N^6^-threonylcarbamoyl adenosine (tRNA modification)
Trp: Tryptophan
xCT (*SLC7A11*): Cystine/Glutamate Antiporter
XRN1: 5′→3′exoribonuclease 1
YRDC: YrdC-domain–containing protein (enzyme for t^6^A formation)
